# Pharmacological Agents Targeting the Cellular Prion Protein

**DOI:** 10.3390/pathogens7010027

**Published:** 2018-03-07

**Authors:** Maria Letizia Barreca, Nunzio Iraci, Silvia Biggi, Violetta Cecchetti, Emiliano Biasini

**Affiliations:** 1 Department of Pharmaceutical Sciences, University of Perugia, 06123 Perugia, Italy; nunzio.iraci@gmail.com (N.I.); violetta.cecchetti@unipg.it (V.C.); 2 Dulbecco Telethon Laboratory of Prions and Amyloids, Centre for Integrative Biology (CIBIO), University of Trento, 38123 Trento, Italy; silvia.biggi@unitn.it; 3 Department of Neuroscience, IRCCS-Istituto di Ricerche Farmacologiche Mario Negri, 20156 Milan, Italy

**Keywords:** cellular prion protein, prion diseases, PrP ligands, pharmacological chaperones

## Abstract

Prion diseases are associated with the conversion of the cellular prion protein (PrP^C^), a glycoprotein expressed at the surface of a wide variety of cell types, into a misfolded conformer (the scrapie form of PrP, or PrP^Sc^) that accumulates in brain tissues of affected individuals. PrP^Sc^ is a self-catalytic protein assembly capable of recruiting native conformers of PrP^C^, and causing their rearrangement into new PrP^Sc^ molecules. Several previous attempts to identify therapeutic agents against prion diseases have targeted PrP^Sc^, and a number of compounds have shown potent anti-prion effects in experimental models. Unfortunately, so far, none of these molecules has successfully been translated into effective therapies for prion diseases. Moreover, mounting evidence suggests that PrP^Sc^ might be a difficult pharmacological target because of its poorly defined structure, heterogeneous composition, and ability to generate different structural conformers (known as prion strains) that can elude pharmacological intervention. In the last decade, a less intuitive strategy to overcome all these problems has emerged: targeting PrP^C^, the common substrate of any prion strain replication. This alternative approach possesses several technical and theoretical advantages, including the possibility of providing therapeutic effects also for other neurodegenerative disorders, based on recent observations indicating a role for PrP^C^ in delivering neurotoxic signals of different misfolded proteins. Here, we provide an overview of compounds claimed to exert anti-prion effects by directly binding to PrP^C^, discussing pharmacological properties and therapeutic potentials of each chemical class.

## 1. Introduction

With few exceptions, proteins evolved their biological function in parallel with the ability to remain soluble under physiological conditions. However, in several pathological situations, specific proteins lose their native fold and acquire a different tertiary and quaternary conformation, clustering into aberrant aggregates. This phenomenon, known as protein misfolding, lays at the root of a wide variety of human diseases, such as neurodegenerative disorders, in which protein aggregation occurs in the brain [1]. Examples include common disorders such as Alzheimer’s and Parkinson’s diseases, or rarer disorders such as amyotrophic lateral sclerosis and prion diseases. Despite the fact that the pathological protein component is different in each neurodegenerative disorder, compelling evidence coming from genetic, biophysical and biochemical studies indicate that misfolded proteins are toxic to neurons. In fact, they often expose regions that are normally buried in the native state, leading to aggregation and aberrant interaction with cellular components such as membranes, proteins, or other macromolecules. These events may negatively affect neuronal homeostasis, for example, by blocking axonal transport, damaging synaptic endings or sequestering essential proteins, ultimately leading to cell death [2]. Possible strategies for tackling protein aggregation include breaking-up aggregates, increasing their degradation, or blocking their formation by stabilizing the native conformation of the monomeric protein precursors. While the first two have largely been explored in the past, the latter is a relatively new concept, and may possibly provide theoretical and technical advantages. For example, although detailed information about the structure of protein aggregates is rarely available, the three-dimensional organization of the monomeric precursors is often well characterized. A particularly meaningful example is represented by prion diseases. These disorders have the peculiarity of manifesting in a sporadic, inherited or transmissible fashion, and are associated with the conformational conversion of the cellular prion protein (PrP^C^), a glycoprotein of uncertain function anchored to the outer surface of the plasma membrane, into a misfolded isoform (called PrP^Sc^) that accumulates in the central nervous system of affected organisms [3]. PrP^Sc^ is a proteinaceous infectious particle (prion), capable of multiplying by directly recruiting native conformers of PrP^C^, and causing their conformational rearrangement into new PrP^Sc^ molecules [4].

The vast majority of experimental strategies aimed at identifying therapeutics for human prion diseases has so far targeted PrP^Sc^, the most direct, pathologically-relevant form of PrP [5]. However, the structure of PrP^Sc^ is poorly defined, and this form is also likely to be heterogeneous in composition and conformation. In fact, one of the most puzzling aspects of prion diseases is the phenomenon of prion strains [6]. It is believed that distinct conformations of PrP^Sc^ may explain the unusually wide spectrum of biochemical, neuropathological and clinical features that characterize prion diseases [7]. Prion strains are of particular relevance for the treatment of prion diseases, as their appearance may cause the acquisition of drug resistance to therapeutic treatments [8,9]. Indeed, a number of previously discovered anti-prion compounds have been shown to act in a strain-specific fashion, a property that severely limits their therapeutic potentials [10,11,12].

**Figure 1:**
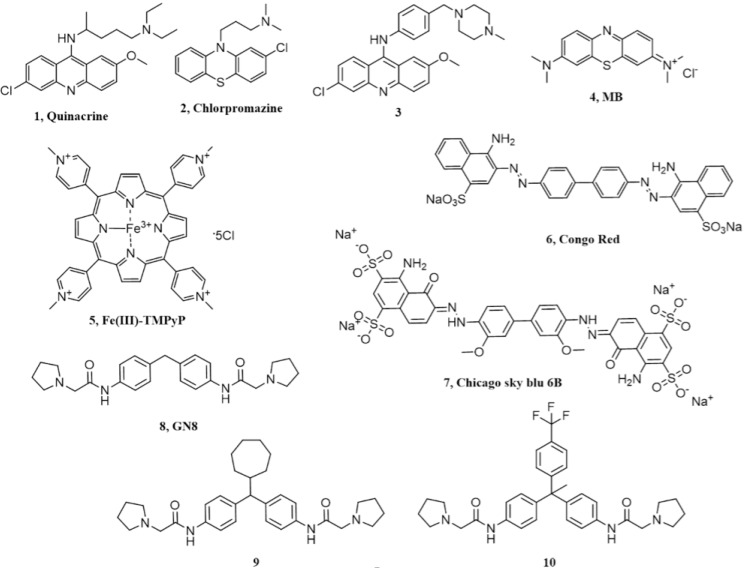

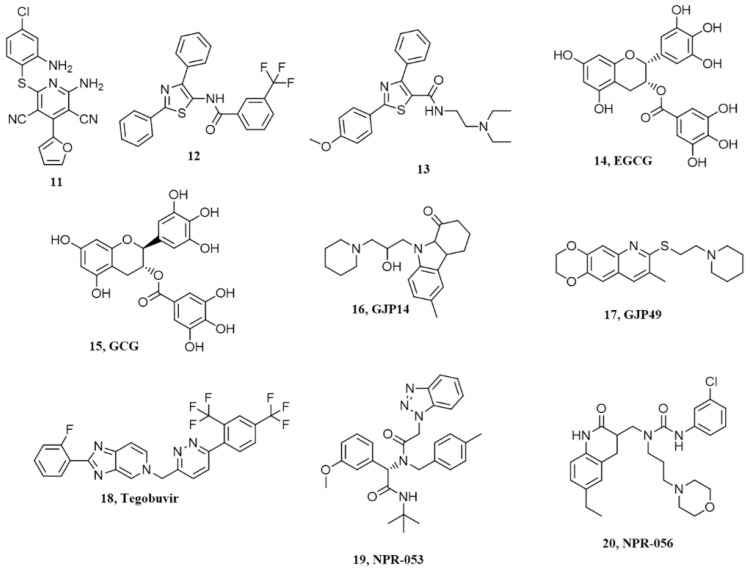
Chemical structures of the different compounds claimed to directly bind PrP^C^.

A possible, perhaps less intuitive strategy to overcome these limitations could be to target PrP^C^, the common substrate of any prion strain replication. The structure of PrP^C^ is known at atomic level resolution, thanks to multiple previous reports employing nuclear magnetic resonance (NMR) or X-ray crystallography [13,14,15]. This provides a convenient ground to carry out rational drug design campaigns. Moreover, from a theoretical standpoint, a molecule binding to PrP^C^ with sufficiently high-affinity might in principle stabilize its folding by reducing the Gibbs free energy. Consequently, the activation energy (∆G) required for the unfolding process will increase proportionally, with the result that the rate of formation of any PrP^Sc^ strain will be kinetically and thermodynamically disfavored. Small molecules acting with such mechanisms are known as pharmacological chaperones. Interestingly, two or more ligands with independent binding sites on PrP^C^ could synergize to completely block the formation of any unfolded form, since the relationship between ∆G and the stability constant of a folded polypeptide chain is exponential. In light of these conclusions, PrP^C^ appears as a convenient molecular target for tackling prion propagation [16]. Is this protein also the right pharmacological target for preventing prion diseases? It is widely agreed that PrP^C^ plays a crucial role in the pathogenesis of prion diseases not only by virtue of its ability to serve as substrate for generation of PrP^Sc^. In fact, it has been reported that genetically depleting neuronal PrP^C^ in mice with established prion infection reverses neuronal loss and progression of clinical signs, despite the continuous production of infectious PrP^Sc^ by surrounding astrocytes [17]. Similarly, the absence of endogenous PrP^C^ renders host brain tissue resistant to the toxic effects of PrP^Sc^ emanating from implanted graft tissue [18]. These data indicate that other toxic species, rather than fully aggregated PrP^Sc^, are responsible for the pathology of prion diseases. This conclusion is consistent with a number of previous reports underscoring the distinction between prion infectivity and prion toxicity [19,20,21,22]. In particular, recent experiments indicate that accumulation of infectivity and neurodegeneration proceed in distinct chronological and mechanistic phases [23]. While infectivity accumulates relatively rapidly, and requires only a minimum expression of PrP^C^, neurodegeneration takes much longer and is directly dependent on the amount of PrP^C^ expressed in the brain. Taken together, these lines of evidence suggest that an unknown PrP conformer, either “on” or “off” pathway to PrP^Sc^, could be the pathological form in prion diseases. These data provide a possible explanation for the evidence that, with few exceptions [12,24], none of the anti-prion compounds identified so far has shown a substantial effect in vivo. In fact, these molecules could disfavor PrP^Sc^ accumulation without hampering the neurotoxicity originating from other toxic conformers. Conversely, stabilizing the folded state of PrP^C^ has the potential to block not only PrP^Sc^ formation and propagation, but also the appearance of any putative toxic conformer. Another potential advantage of targeting PrP^C^ arises from recent observations indicating that PrP^C^ may exert a toxicity-transducing activity upon binding to PrP^Sc^, as well as to various disease-associated, misfolded oligomeric assemblies, such as those formed by the amyloid β (Aβ) peptide, or by the protein alpha-synuclein, linked to Alzheimer’s and Parkinson’s diseases, respectively [25,26,27,28,29]. Importantly, mice depleted for PrP expression develop normally, with subtle phenotypic changes appearing only later in life, thus suggesting that pharmacological decrease of PrP^C^ function could produce little side effects. This conclusion is also supported by the recent identification of loss-of-function PrP alleles in healthy subjects [30]. Overall, these data support the potential value of targeting PrP^C^, as this approach may provide therapeutic benefits not only for prion diseases, but possibly also for other neurodegenerative disorders. In this manuscript, we review the main chemical classes reported to act against prion replication in a PrP^C^-directed fashion, focusing our discussion on molecules for which binding constant (K_D_), structural information and anti-prion half-maximal effective concentration (EC_50_) have experimentally been determined (Figure 1). 

## 2. Acridine and Phenothiazine Derivatives

**Figure 2:**
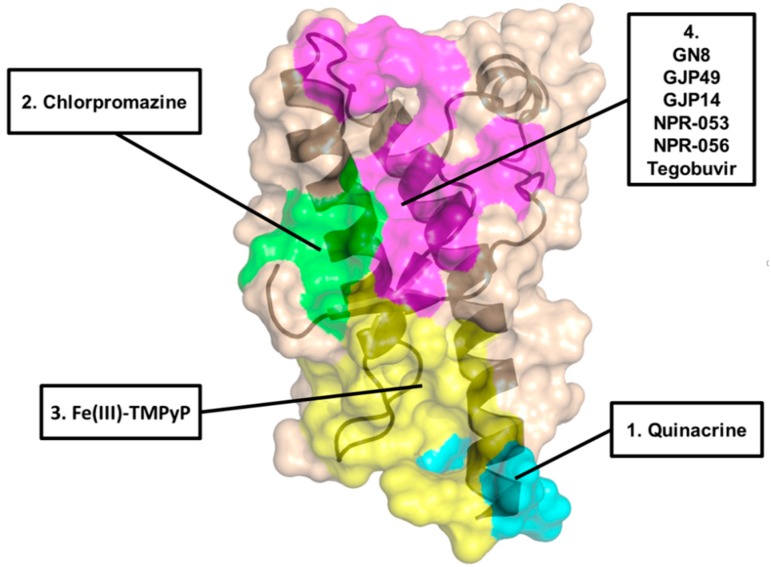
Visualization of the proposed binding regions for the different PrP^C^ ligands (indicated).

Tricyclic derivatives of acridines (compound **1** in Figure 1, quinacrine) and phenothiazines like chlorpromazine (compound **2** in Figure 1) were initially reported to be promising candidates for the treatment of prion diseases [31,32]. Indeed, these drugs have already been used in humans for many years, and are known to cross the blood–brain barrier, thus giving hope to their repurposing for prion diseases. The antimalarial agent quinacrine and the antipsychotic drug chlorpromazine showed inhibition of PrP^Sc^ formation in prion-infected N2a cells, with EC_50_ values of ~0.3 µM and ~3 µM, respectively. The acridine derivative quinacrine deserves particular attention, as it showed better potency in cell cultures, and was tested in human trials for prion diseases (more extensively than chlorpromazine, which was tested only in combination with the antimalarial agent). Quinacrine enantiomers showed stereoselectivity against prions, with the (S)-quinacrine exhibiting superior activity in eradicating PrP^Sc^ from cells [33]. Unfortunately, despite the promising in vitro profile, no beneficial effects were observed in vivo, using prion-infected rodent models of prion disease [34,35]. In addition to animal models, the activity and safety of quinacrine was assessed in clinical trials in human Creutzfeldt–Jakob disease (CJD) patients, but no effects were observed either on survival at the two-month time point or on the clinical course of the disease [36,37]. Pharmacokinetic studies unveiled that free quinacrine concentration in the brain reached only ~1 µM, which is a lower value than the cellular EC_50_ observed in vitro [11,38]. These results highlighted the difficulty of translating results obtained by in vitro or cell-based methods to the clinical context. The lack of clinical efficacy of quinacrine against CJD was mainly attributed to metabolic instability, scarce accumulation of the drug into the brain due to active efflux by P-glycoprotein (P-gp) and the formation of drug resistant prion strains [11]. Original studies suggested that the anti-prion activity of quinacrine was directly connected to its ability to modify the lysosomal environment, causing improved clearance of PrP^Sc^ [31]. However, later studies reported that quinacrine binds to the globular domain of human recombinant PrP (residues 121–230), as observed by NMR spectroscopy. Tyr225, Tyr226, and Gln227 of helix 3 (H3) were identified as key residues in such ligand–protein interaction (region 1 in Figure 2) [39]. Of note, these experiments were conducted at very high concentrations, and the obtained dissociation constant of quinacrine (K_D_ = 4.6 mM) was about four orders of magnitude higher than its cellular EC_50_ value (required to clear PrP^Sc^ from prion-infected cells in vitro). Similar data (K_D_ ~ 1 mM) were obtained in another study where quinacrine binding to recombinant human PrP was analyzed by surface plasmon resonance (SPR) [40], although other SPR studies reported the ability of quinacrine to bind human recombinant PrP with a K_D_ of 15 µM [41]. In another report, dynamic light scattering studies and circular dichroism (CD) measurements suggested that quinacrine binding induces a conformational change in PrP, disfavouring PrP^Sc^ formation [42]. It is worth noting that the potential of quinacrine as a prion inhibitor has stimulated great interest in the 9-aminoacridine family as therapeutic candidates for prion diseases, and intensive research efforts have been spent on the synthesis, biological evaluation and structure–activity relationship (SAR) studies of quinacrine derivatives [43,44,45,46]. In particular, the nature of the aliphatic side-chain on 9-amino group of the tricyclic scaffold was found to be one key feature for enhancing binding affinity to PrP, PAMPA permeability and inhibition of PrP^Sc^ accumulation. As an example, a quinacrine derivative (compound **3** in Figure 1) showed improved anti-prion activity, as compared to the parent compound, across different prion-infected murine cell models (ScN2a, N167, F3). In addition, this compound exhibited stronger binding affinity by SPR, and seemed to be a weaker substrate for P-gp [46]. However, more recent SPR- and NMR-based studies have highlighted a non-specific binding interaction of quinacrine to PrP^C^, reiterating the original observation that its mode of action involves PrP-independent mechanisms [47,48].

Similarly to quinacrine, the direct binding of phenothiazine derivative chlorpromazine to PrP^C^ was originally investigated by NMR [39] and SPR [41], showing a weaker interaction with recombinant PrP, as compared to quinacrine. A subsequent study based on NMR and X-ray crystallography (PDB ID 4MA8) reported a precise binding site of phenothiazines on PrP^C^, located in a hydrophobic pocket formed by helix-2 (H2) and the two anti-parallel β-sheets (S1 and S2; region 2 in Figure 2) [49]. The data also indicated that an unexpected intramolecular reorganization of the N-terminal, unstructured tail of PrP^C^ around the C-terminal domain, through the formation of a hydrophobic anchor, directly suggesting a mechanism by which phenothiazines may act as pharmacological chaperone of PrP^C^. Unfortunately, the study did not provide an affinity value for the binding of phenothiazines to PrP^C^. Such value was instead precisely defined in the following report, employing SPR and dynamic mass redistribution (DMR) [50]. The results confirmed original observations indicating a weak interaction of chlorpromazine to PrP^C^, with an estimated K_D_ higher than 400 µM, compatible with data collected in the original study [49], which employed millimolar concentrations of chlorpromazine to carry out NMR and X-ray crystallography experiments. A K_D_ value in the high micromolar concentration range is incompatible with the reported anti-prion effects of chlorpromazine in cells, indicating that its mode of action is independent from direct PrP binding [50]. Moreover, chlorpromazine also failed to inhibit prion replication in vitro (by the protein misfolding cyclic amplification reaction, PMCA), as instead it would be expected for a pharmacological chaperone of PrP^C^. Interestingly, the same study reported compelling evidence indicating that the mechanism of action underlying the anti-prion effect of chlorpromazine is related to the previously known ability of the compound to inhibit clathrin-mediated endocytosis, leading to decreased levels of PrP^C^ at the cell surface. Consistent with this conclusion, two inhibitors of dynamins, proteins involved in the regulation of the scission of membrane vesicles, and recently reported to be targeted by chlorpromazine [51], mimicked PrP^C^-relocalizing effects, and blocked the replication of two different prion strains in cell cultures [50]. An additional recent work provided evidence for a chlorpromazine-induced redistribution of PrP^Sc^ from the endocytic-recycling pathway to the lysosomal compartment, an effect that could be the direct consequence of the relocalization of PrP^C^ from the cell surface [52].

Methylene Blue (MB, compound **4** in Figure 1), a phenothiazine derivative, has been shown to affect the kinetics of PrP oligomerization by binding to a surface cleft on PrP^C^ [53]. Using size exclusion chromatography, static light scattering, differential scanning calorimetry and transmission electron microscopy, the authors studied the influence of methylene blue on the oligomerization and fibrillation of human, ovine and murine recombinant PrP, observing a decrease in oligomerization kinetics and overall levels. NMR experiments mapped MB binding sites in a surface cleft delimited by residues belonging to S1-H1 and H2-H3 loops, and H1, H2 and H3 helices (residues Asn146, Asn156, Tyr160, Lys188, Thr191, Val192, Thr194, Thr195, Gln215). Of note, MB has been investigated as potential therapeutic agent in other proteinopathies [54,55,56,57], which is consistent with the number of potential applications that have been tested for this compound, likely reflecting its ability to engage non-specific interactions with a broad range of proteins.

## 3. Cyclic Tetrapyrroles

Cyclic tetrapyrroles, planar aromatic ring systems coordinating metal ions and bearing pendants of different chemical nature, were originally found to be effective in prion-infected cells, and later claimed to act by directly binding to PrP^C^ [58,59]. In particular, by employing isothermal titration calorimetry (ITC), the cationic tetrapyrrole Fe(III)-TMPyP (compound **5** in Figure 1) was shown to bind human recombinant PrP in the C-terminal, globular domain (K_D_ = 4.52 µM), which was consistent with its cellular EC_50_ of 1.6 µM in cells (as tested in rocky mountain laboratory, RML-infected PK1 cells) and the range of concentrations (1–11 µM) active in the protein-misfolding cyclic amplification (PMCA) reaction [48]. NMR studies allowed the identification of the binding site of Fe(III)-TMPyP on human PrP, with key interacting residues clustered at the C terminus of H3 and in the loop between residues 160 and 180 (region 3 in Figure 2). Importantly, Fe(III)-TMPyP, or highly similar porphyrins, also showed the ability to inhibit the cytotoxic activity of a mutant PrP carrying a deletion in the central region (Δ105–125), abrogated the PrP^C^-mediated synaptotoxic effects of Aβ oligomers in primary hippocampal neurons, and significantly prolonged survival time in prion-infected mice [60,61]. Unfortunately, the therapeutic potentials of porphyrins like Fe(III)-TMPyP is dampened by their poor pharmacokinetic properties, such as possible non-specific interactions with plasma proteins, and unlikelihood to cross the blood–brain barrier [62]. However, as assayed by in vitro and cell-based tests, these compounds appear as the most effective pharmacological chaperones of PrP^C^, and have already been employed to gain insights into the physiological activity of PrP^C^, and its functional connection to neurodegenerative pathways. Performing extensive pharmacokinetic profiling of this class of molecules, coupled to chemical optimization efforts and/or innovative ways of delivery to the central nervous system, could provide effective therapeutic strategies for prion diseases, and possibly other neurodegenerative disorders linked to the toxicity-transducing activity of PrP^C^.

## 4. Diazo Dyes

The diazo dye Congo red (compound **6** in Figure 1) was found to possess anti-prion activity in cells and in vivo, using scrapie-infected golden Syrian hamsters [63,64,65,66,67]. In particular, Congo red prevented the formation and accumulation of PrP^Sc^ in neuroblastoma cells with an EC_50_ of about 0.015 µM. The binding of Congo red to human recombinant PrP was investigated by SPR, and showed a K_D_ value of 1.6 µM [40]. However, other studies reported that, in physiological conditions, the molecule binds non-specifically to PrP^C^ as an aggregated polyanion [47]. Congo Red itself has a number of shortfalls, such as non-specific interactions with various macromolecules, self-polymerization, toxicity and poor permeability through BBB. For this reason, several Congo red derivatives were designed and synthesized to improve the pharmacological profile of the compound, and a number of analogues showed anti-prion effects at nanomolar concentrations, even though no information about their possible interaction with PrP^C^ was reported [68,69,70].

## 5. Chicago Sky Blue 6B

This molecule emerged from a screen of 1200 approved drugs and pharmacological tool compounds (Prestwick Chemical Library) based on a fluorescence polarization (FP) assay, and aimed at identifying compounds capable of inhibiting the binding of Aβ oligomers to PrP^C^ [71]. Chicago Sky Blue 6B (compound **7** in Figure 1) was identified as the best-ranked candidate, with EC_50_ values of 0.41 µM and 19.7 µM in FP and ELISA assays, respectively. ITC experiments confirmed that Chicago Sky Blue 6B is able to interact with human recombinant PrP, with a K_D_ value of 0.55 µM. Importantly, the compound did not bind a PrP construct containing only residues 119–231, indicating that its binding site lies within the N-terminal, unstructured tail of the protein. Since Aβ oligomers are known to bind PrP in the same region, the data suggested that Chicago Sky Blue 6B may act by a mechanism of direct competition. Of note, Chicago Sky Blue 6B also showed anti-prion effects in RML-infected N2a cells, with EC_50_ values in the low micromolar range, and in absence of evident cytotoxicity. At the time this manuscript was prepared, no other studies have employed Chicago Sky Blue 6B in the context of prion diseases.

## 6. Diphenylmethane Derivatives

A compound known as GN8 (compound **8** in Figure 1) emerged from an in silico, dynamics-based drug screen of ~320,000 compounds aimed at directly identifying pharmacological chaperones for PrP^C^ [72]. In vitro validation studies estimated the affinity of GN8 for recombinant, mouse PrP in the low micromolar range (KD ~ 5 µM). Heteronuclear NMR and molecular modeling mapped the PrP binding region of GN8 at the C-terminal domain, particularly involving residues N159 and E196 (region 4 in Figure 2). Furthermore, the authors employed CD in a thermal-denaturation assay to confirm that the binding of GN8 stabilizes the PrP^C^ conformation significantly (ΔΔH = 6.7 kcal/mol). Biological validation showed that GN8 efficiently inhibits prion replication in cells, with an estimated EC_50_ of ~1.35 µM. Importantly, GN8 was also found to prolong the survival of prion-infected mice, thus confirming the effective anti-prion activity of this molecule. Subsequent studies focused on the synthesis and evaluation of anti-prion effects for a series of GN8 analogues with the main objective of generating a SAR profile [73]. Two derivatives (compounds **9** and **10** in Figure 1) were found to be approximately three times more potent than the parent compound, with EC_50_ values around 0.5 µM, in absence of detectable toxicity. CD-coupled thermal-denaturation assays indicated that one of these molecules significantly stabilized recombinant PrP, with a degree of stabilization by this ligand approximately doubled, as compared with that of GN8 (ΔΔH = 14.2 kcal/mol). Binding was also confirmed by SPR. According to these data, GN8 and its derivatives appear as promising pharmacological chaperones of PrP^C^. However, it is worth noting that two subsequent studies failed to confirm binding of GN8 to mouse or human recombinant PrP, using a battery of biophysical techniques [48,60]. Such experimental discrepancy is currently unresolved.

## 7. Pyridine Dicarbonitriles

Four pyridine dicarbonitrile analogues, originally identified as anti-prion compounds in prion-infected cells [74], were later tested for their direct interaction with PrP^C^ using SPR [41]. One derivative (compound **11** in Figure 1) showed anti-prion activity (EC_50_ values ~ 20 µM) and detectable binding to recombinant PrP. This observation justified the following efforts to generate small libraries of pyridine dicarbonitrile derivatives, which were then tested by SPR for binding to PrP^C^, and in cellular assays to evaluate anti-prion activity [75,76]. Unexpectedly, no direct correlation was observed between binding to PrP^C^ and anti-prion efficacy, with the most potent anti-prion pyridine dicarbonitrile showing either weak or no binding to PrP^C^. Collectively, these data suggested that pyridine dicarbonitrile likely inhibit prion replication in a PrP^C^-independent fashion.

## 8. Diarylthiazoles

The same team originally involved in the study on the dicarbonitrile derivatives also reported the synthesis and screening of 2,4-diarylthiazole-based compounds as potential anti-prion agents [77,78]. The authors stated that original 2,4-diarylthiazole scaffold was identified as a PrP ligand through a virtual screening campaign, although details of such screening were not described. SPR was then employed to test the binding of several derivatives to mouse or human recombinant PrP. Only one compound (compound **12** in Figure 1) showed a high-affinity interaction with PrP. All the molecules were also tested in prion-infected SMB cells, but once again no correlation was found between PrP binding and anti-prion activity in cells. A second series of reverse amide 2,4-diarylthiazole-based anti-prion compounds was later reported in a following study. The molecules were first tested for prion inhibition in SMB cells and then evaluated for binding to recombinant PrP, as assayed by SPR. Among the compounds active in cells, one derivative (compound **13** in Figure 1) (EC_50_ = 4 µM) also showed affinity for PrP, although a careful evaluation of SPR data suggested the possibility of a non-specific interaction. Overall, these studies highlighted a general lack of correlation between anti-prion activity and PrP binding for 2,4-diarylthiazole-based compounds, suggesting that other PrP-independent modes of action account for the anti-prion effects of this chemical class.

## 9. Natural Polyphenols

In search of small molecules able to interfere with prion propagation, another study screened a collection of natural compounds with proven activity against amyloid formation in vitro [79,80]. The major polyphenols component of green tea, i.e., epigallocatechin gallate (EGCG, compound **14** in Figure 1) and its stereoisomer gallocatechin gallate (GCG, compound **15** in Figure 1), showed anti-prion activity in prion-infected N2a cells. The direct interaction of EGCG with recombinant PrP (residues 90–232) was experimentally tested by ITC, showing a strong affinity (K_D_ = 0.13 µM) and a remarkable stabilization effect (ΔH of −43 KJ). Further experiments on the effect of EGCG binding revealed an unexpected destabilization effect of the compound on the native conformation of PrP^C^, inducing its rapid transition into detergent-insoluble species, which were rapidly degraded intracellularly. The authors also observed that the anti-prion activity depended on the gallate side chain and the three hydroxyl groups of the trihydroxyphenyl side chain. Unfortunately, a subsequent study characterized the binding properties of EGCG to PrP^C^ by SPR and NMR, concluding that the compound binds to the protein in a non-specific fashion [47]. These results dampened the enthusiasm for the treatment of prion diseases with EGCG-like polyphenols.

## 10. Miscellanea

Several structurally diverse compounds identified by virtual screening campaigns on the proposed binding pocket for GN8 have been claimed to be specific PrP^C^ ligands, capable of acting as chemical chaperones. In 2009, a virtual screening study led to the selection of 205 commercially available compounds to be evaluated for their effects on the PrP^C^ conversion process [81]. Ex vivo-experiments identified 24 non-cytotoxic molecules that significantly inhibited prion replication in GT-FK cells, at a concentration of 10 µM. To further elucidate their mechanism of action, the authors measured the binding affinity for recombinant PrP by SPR, and then compared anti-prion activity in cells with affinity values. Eleven compounds were classified as PrP-directed anti-prion compounds; for example, for a molecule named GJP14 (compound **16** in Figure 1), the authors reported an EC_50_ = 8.54 µM [82]. Compounds GJP14 and GJ49 (compound **17** in Figure 1) were further characterized for their binding properties by SPR and NMR (for example, for GJ49 K_D_ = 50.8 µM), showing a ligand-binding pocket in the C-terminal, globular domain of PrP^C^ (region 4 in Figure 2) [47].

A related study performed a 3D pharmacophore-based virtual screen of an in-house chemical library, and selected 37 potential anti-prion compounds to be assessed by cell-based and SPR-based assays [83]. The results identified a molecule named BMD42-29 (a benoxazole derivative whose structure was not disclosed) as the best hit among the screened molecules, with an EC_50_ value against prion replication in cells in the low micromolar range (<5 µM). Of note, in prion-infected N2a cells, the compound did not produce a marked reduction in total PrP levels. SPR experiments revealed that BMD42-29 had strong binding affinity to PrP^C^ (K_D_ = 21.5 µM), with kinetic rates characterized by rapid association and slow dissociation constants. The predicted binding mode of BMD42-29 was located in the same pocket of GN8, and was characterized by two hydrogen bonds with Asn159 and Glu196, and hydrophobic interactions with Leu130 and Arg156. The author concluded that BMD42-29 may act by stabilizing PrP^C^, thus inhibiting its pathological conformational change to PrP^Sc^. In 2016, another group built a platform called “NAGARA”, aimed at unifying docking simulation, molecular dynamics and quantum chemistry to perform large-scale screening of commercially available compounds [84]. One hundred hits predicted in silico to bind PrP^C^ were subjected to cell-based validation to evaluate anti-prion effects. Tegobuvir (previously known as an anti-hepatitis C agent, compound **18** in Figure 1) emerged as one of the most promising candidates, with an estimated EC_50_ of 1.7 µM, as assayed in immortalized neuronal mouse cells persistently infected with the human Fukuoka-1 prion strain. The molecule also showed detectable binding to PrP^C^ in the low micromolar range (K_D_ = 19 µM, region 4 in Figure 2). In the same year, by coupling docking simulations of a large virtual library (~200K compounds) and binding interaction analyses, another group reported the identification of 96 novel small molecules capable of binding PrP^C^ in the same pocket of GN8 [85]. The ability of the in silico-predicted hits to target PrP^C^ was evaluated by SPR and thermal shift assay (TSA), whereas their anti-prion effects were estimated using persistently infected cells and animal models of prion diseases. Compounds NPR-053 (compound **19** in Figure 1) and NPR-056 (compound **20** in Figure 1) emerged as the most promising candidates, in light of their ability to reduce PrP^Sc^ levels in cultured cells, with EC_50_ values of 7.68 µM and 3.72 µM, respectively. Both SPR and TSA provided evidence for a direct binding of both compounds to PrP^C^ (region 4 in Figure 2), with NPR-053 inducing the strongest stabilization effect on PrP^C^ native folding (ΔT_m_ = 2.69 °C). All of these compounds represent promising candidate pharmacological chaperones for PrP^C^, although further experimental validation is needed before considering them as promising therapeutic agents for prion diseases.

## 11. Conclusions

Mounting evidence indicates that the accumulation of PrP^Sc^ alone could not account for the wide spectrum of neurotoxic events occurring in prion diseases. Instead, an unexpected role for PrP^C^ as toxicity–transducer receptor for PrP^Sc^ and other disease-associated misfolded oligomeric assemblies, such as Aβ and alpha-synuclein, has raised great interest for targeting this protein pharmacologically. In this manuscript, we reviewed previous efforts to identify PrP^C^-directed compounds, taking into account limitations and reproducibility of each experimental attempt. A number of chemical scaffolds, identified by combining computational methods with biochemical, biophysical and cell-based assays, have been claimed to exert anti-prion effects by targeting PrP^C^ (Table 1). Some of these molecules, such as the cationic tetrapyrrole Fe(III)-TMPyP, provide a proof-of-principle for targeting PrP^C^ pharmacologically. Others, such as chlorpromazine, reveal unexpected mechanisms to counteract prion replication by lowering cell surface PrP^C^. However, the vast majority of compounds show inconsistencies between affinity for PrP^C^ and biologically-active concentrations, low binding specificity, and/or lack of reproducibility. At the moment, none of these molecules appear as immediate candidates for clinical testing in the near future. Moreover, the great deal of negative data eventually provide further support to the notion that the vast majority of anti-prion molecules identified so far exert their activity through unknown targets, or by altering the homeostasis of PrP^C^, rather than binding the protein directly. What could be the reason for such a lack of success in identifying small ligands of PrP^C^? We believe the answer to this question may lie in a few, non-mutually exclusive possibilities. First, the screening techniques employed so far (e.g., in silico approaches coupled to biophysical assays) could have been inadequate for effectively identifying PrP^C^-directed molecules. Moreover, most of the approaches reviewed in this manuscript relied on recombinant PrP for testing the binding of small molecules, while physiological, post-translational modifications of the protein (sugar and lipid moieties) may heavily influence ligand binding. It is also possible that a single PrP^C^ ligand will never be truly effective in preventing prion replication, since its stabilization effect on PrP^C^ folding could be counteracted by the strong affinity of PrP^Sc^ for its substrate. In this scenario, testing the combination of two or three ligands binding PrP^C^ in distinct pockets may produce the expected anti-prion effects. Ultimately, it is also possible that PrP^C^ simply lies among the proteins that can be classified as “undraggable”. We like to believe that the latter conclusion will soon be refuted by direct experimental evidence.

## Figures and Tables

**Table 1:** Summary of main chemical scaffolds reported to exert anti-prion effects by directly targeting PrP^C^.

Chemical Scaffold	Compound (Figure 1)	K_D_ *	EC_50_ **	Effect In Vivo ***	Conclusions
**Acridine derivatives**	**1**	~1mM	~0.3 µM	Not significant	Primary effects are PrP-independent
**Phenothiazine derivatives**	**2**	>400 µM	~3 µM	Not significant	Likely acting by inducing PrP^C^ re-localization from the cell surface
**Tetrapyrroles**	**5**	4.52 µM	1.6 µM	Prolongation of survival time in prion-infected mice	Low specificity and possible poor pharmacokinetics
**Diazo dyes**	**6**	1.6 µM	0.015 µM	Not available	Low specificity
**Chicago sky blue 6B**	**7**	0.55 µM	Low µM	Not available	Need confirmation
**Diphenylmethane derivatives**	**8**	5 µM	1.35 µM	Prolongation of survival time in prion-infected mice	PrP^C^ binding not reproduced in some study
**Pyridine Dicarbonitriles**	**11**	~20µM	18.6 µM	Not available	No correlation between anti-prion activity and binding to PrP^C^
**Diarylthiazoles**	**13**	3.8 µM	4 µM	Not available	No correlation between anti-prion activity and binding to PrP^C^
**Natural polyphenols**	**14**	0.13 µM	-	Not available	Possible non-specific interaction with PrP^C^
**Miscellanea**	**17**	50.8 µM	Not available	Not available	Need confirmation
	**20**	19 µM	3.72 µM	Not available	Need confirmation

* Reported affinity for PrP^C^; ** Anti-prion activity measured in cell cultures; *** Tested in prion-infected rodent models and/or human patients.
